# Measurement of the Distance between the Mitral Annulus and the Left Circumflex Coronary Artery Using Multiplanar Reconstruction of Intraoperative Transoesophageal Echocardiography Images

**DOI:** 10.1093/icvts/ivag022

**Published:** 2026-01-21

**Authors:** Yuki Kuroda, Yoshiharu Soga, Takehiko Matsuo, Shinichi Tsumaru, Keisuke Hakamada, Yuki Wada, Yuta Kitagata, Ryo Imada, Akira Marui, Nobuhisa Ohno

**Affiliations:** Department of Cardiovascular Surgery, Kokura Memorial Hospital, Kitakyushu, 803-8555, Japan; Department of Cardiovascular Surgery, Graduate School of Medicine, Kyoto University, Kyoto, 606-8507, Japan; Department of Cardiovascular Surgery, Kagoshima University, Kagoshima, 890-8520, Japan; Department of Cardiovascular Surgery, Kokura Memorial Hospital, Kitakyushu, 803-8555, Japan; Department of Cardiovascular Surgery, Kokura Memorial Hospital, Kitakyushu, 803-8555, Japan; Department of Cardiovascular Surgery, Kokura Memorial Hospital, Kitakyushu, 803-8555, Japan; Department of Cardiovascular Surgery, Kokura Memorial Hospital, Kitakyushu, 803-8555, Japan; Department of Cardiovascular Surgery, Kokura Memorial Hospital, Kitakyushu, 803-8555, Japan; Department of Cardiovascular Surgery, Kokura Memorial Hospital, Kitakyushu, 803-8555, Japan; Department of Cardiovascular Surgery, Kokura Memorial Hospital, Kitakyushu, 803-8555, Japan; Department of Cardiovascular Surgery, Kokura Memorial Hospital, Kitakyushu, 803-8555, Japan

**Keywords:** mitral annular disjunction, mitral valve surgery, transoesophageal echocardiography

## Abstract

**Objectives:**

We aimed to describe the anatomical distance between the mitral annulus and the left circumflex coronary artery (LCX) using multiplanar reconstruction (MPR) of transoesophageal echocardiography (TEE) images and to investigate its association with mitral annular disjunction (MAD).

**Methods:**

A single-centre retrospective cohort study included 54 patients who underwent mitral valve repair for mitral regurgitation between January 2020 and July 2021. We measured the distance between the mitral annulus and the LCX (ML distance) using MPR of intraoperative TEE images. As an exploratory analysis, we compared the ML distance between patients with MAD (group D: *N* = 11) and those without (group N: *N* = 43).

**Results:**

The LCX was closest to the mitral annulus at 70-90 degrees counterclockwise from the anteroposterior axis. No cases of LCX injury were observed. MAD was most frequently observed at P1, and all patients in group D had disjunction at P1. The minimum ML distance was significantly shorter in group D than in group N (3.2 [1.1] mm in group D, and 4.9 [2.1] mm in group N). Overall, the ML distance was shorter in group D than in group N, and was significantly shorter at 70-100 degrees.

**Conclusions:**

MPR of intraoperative TEE images is a less invasive and useful tool to detect patients with a short ML distance. The area of the closest distance from the mitral annulus to the LCX is near the anterolateral commissure, especially in patients with MAD.

## Graphical abstract

**Figure:**
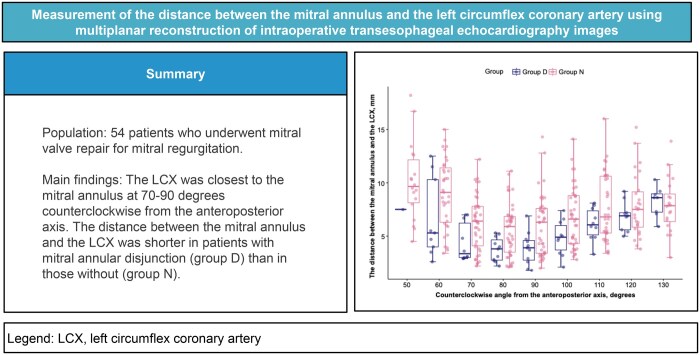

## INTRODUCTION

One of the complications associated with mitral valve surgery is injury to the left circumflex coronary artery (LCX).^1^ The LCX is close to the mitral annulus in the vicinity of the anterolateral commissure and P1.^2^

Detection of the anatomical relationship between the LCX and the mitral annulus using three-dimensional computed tomography (3D CT) has been reported.^3^ We have been measuring the distance between the mitral annulus and the LCX (ML distance) using multiplanar reconstruction (MPR) of the intraoperative transoesophageal echocardiography (TEE) images, which we routinely perform during cardiovascular surgery. When the close proximity of the LCX to the mitral annulus is identified by TEE, the surgeons are alerted. During the process of evaluating the ML distance using TEE, we frequently observed that the ML distance was particularly short in patients with mitral annular disjunction (MAD).

MAD is a specific anatomical abnormality associated with mitral valve prolapse, characterized by a separation between the left atrium/mitral valve annulus and left ventricular myocardium.^4^^,^^5^ MAD was reported to be associated with annular contractile dysfunction and non-sustained ventricular tachycardia.^6^

We aimed to describe the anatomical distance between the mitral annulus and the LCX using MPR of TEE images and to investigate its association with MAD.

## METHODS

### Ethical statement

This single-centre retrospective cohort study was approved by the Institutional Review Board of Kokura Memorial Hospital (21100601; approval date: October 6, 2021). Because of the retrospective nature of the study, the requirement for written informed consent was waived. The collection and storage of biological materials and data in this study were conducted in accordance with the principles of the WMA Declaration of Taipei.

### Study design

We measured the anatomical parameters of the mitral valve and the ML distance in 54 patients who underwent mitral valve repair for degenerative mitral regurgitation at Kokura Memorial Hospital between January 2020 and July 2021. Combined procedures such as aortic valve surgeries, tricuspid valve surgeries, and arrhythmia surgeries were included. We excluded cases where the mitral annulus was extremely large, and the LCX was outside the visible range of three-dimensional TEE. Furthermore, as an exploratory analysis, we compared the distance based on the presence or absence of MAD: patients with MAD (group D, *N* = 11, 20%) and patients without MAD (group N, *N* = 43, 80%). Patient characteristics and operative data were obtained from medical records. Baseline characteristics and anatomical parameters obtained from transthoracic and transoesophageal echocardiography were compared between the groups. Also, we compared the ML distance according to the coronary bed anatomy.

### Measurement methods

**Figure 1.**
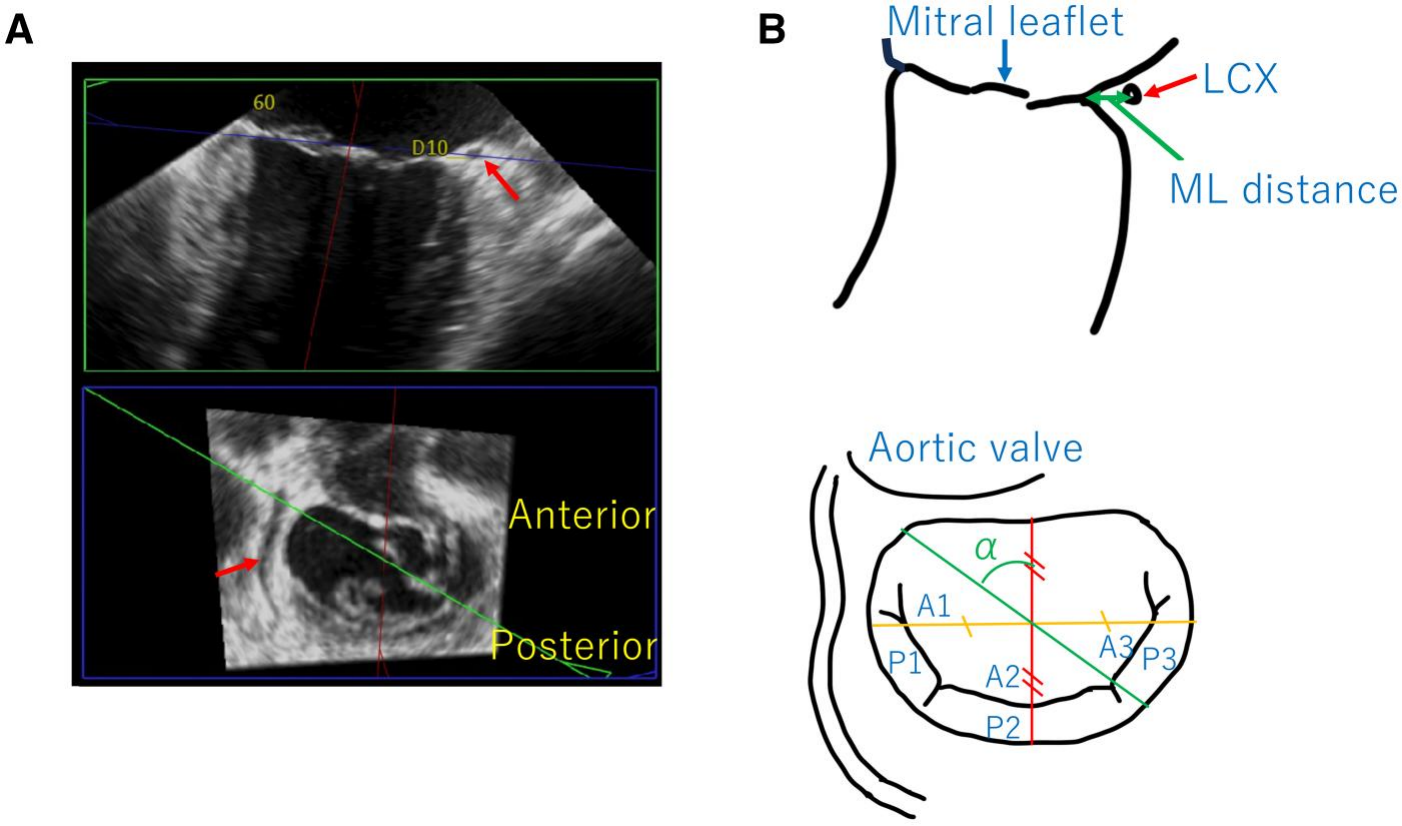
MPR Images Obtained from TEE Images (A) and Their Schematic Diagram (B). (A) The upper image is a long-axis image of the lower image cut by the cross-section of the green line. Red arrows show the LCX. (B) The upper image is a long-axis image of the lower image cut by the cross-section of the green line. The ML distance is the distance between the mitral annulus and the LCX. α is the counterclockwise angle from the anteroposterior axis. MPR, multiplanar reconstruction; TEE, transoesophageal echocardiography; LCX, left circumflex coronary artery

**Figure 2.**
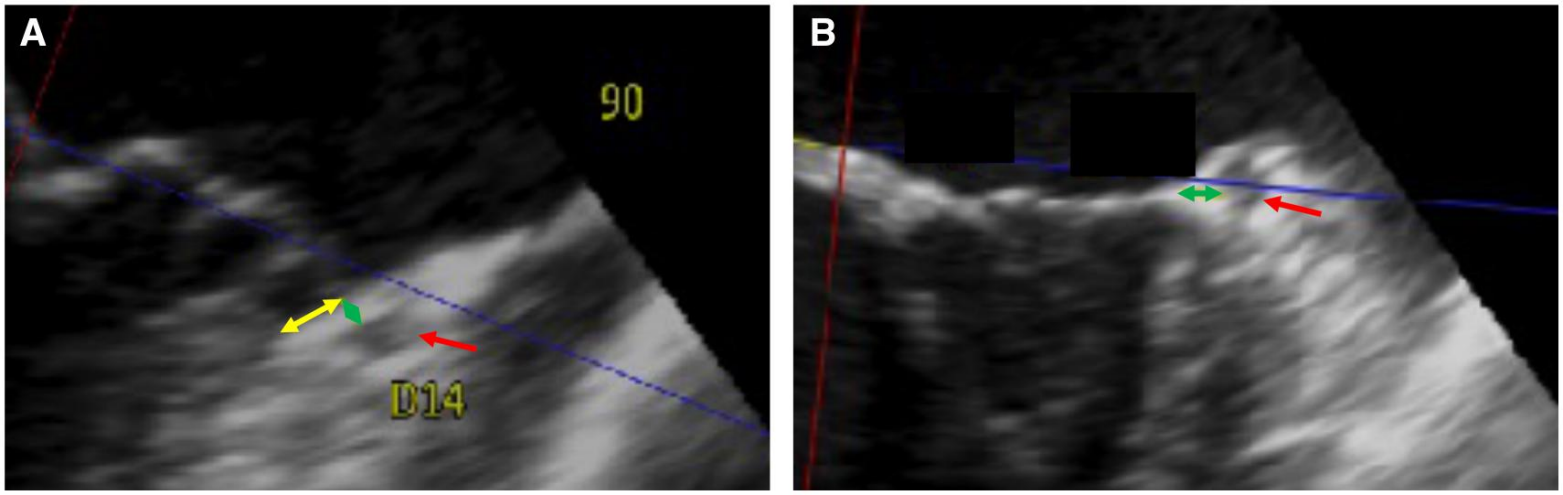
Long Axis MPR Images at 90 Degrees Counterclockwise Angle from the Anteroposterior Axis in Patients with MAD (A) and Patients without MAD (B). The yellow arrow shows MAD. Red arrows show the LCX. Green arrows show the distance between the mitral annulus and the LCX. MPR, multiplanar reconstruction; MAD, mitral annular disjunction; LCX, left circumflex coronary artery

Intraoperative TEE was performed routinely. We performed TEE using EPIC CVx (Philips, Amsterdam, The Netherlands). We confirmed the LCX in the mitral commissural view and captured the 3D images with the Full Volume mode. Captured images were post-processed using commercially available software (3DQ, Philips, Amsterdam, The Netherlands). We adjusted one of the MPR cross-sections to the height of the mitral valve attachment in the end-systolic phase, and adjusted the other two cross-sections so that they became cross-sections perpendicular to the previous cross-section and included the anteroposterior and transverse diameters of the mitral annulus. Using the intersection of the anteroposterior and transverse diameter cross-sections as the centre, we rotated the cross-section perpendicular to the mitral valve attachment cross-section and measured the ML distance. Measurements of the ML distance were taken in sections every 10 degrees (50-130 degrees) counterclockwise from the anteroposterior axis of the mitral valve (**Figures 1 and 2**). We defined MAD as >2 mm of separation between the left atrium/mitral valve annulus and left ventricular myocardium on the cross-section perpendicular to the mitral valve attachment cross-section. All measurements were performed by a single observer with over 10 years of experience in intraoperative TEE and measurements of the mitral valve.

### Statistical analysis

Categorical variables were presented as numbers (percentages) and were compared using Fisher’s exact test. Continuous variables were presented as mean (standard deviation) or median (interquartile range [IQR]) and were compared using the *t*-test, 1-way ANOVA, or the Wilcoxon rank-sum test. Effect sizes and 95% confidence intervals were estimated using the linear regression models. All statistical analyses were conducted using R statistical software version 4.4.1 (R Core Team (2024). _R: A Language and Environment for Statistical Computing_. R Foundation for Statistical Computing, Vienna, Austria. https://www.R-project.org/.). All reported *P* values were two-tailed, and *P* values less than .05 were considered statistically significant.

## RESULTS

### Patient characteristics

Baseline characteristics, including age, sex, body surface area (BSA), and underlying diseases, were not significantly different between the groups (**Table 1**). Left ventricular function was also not significantly different between the groups.

**Table 1.** Baseline and Procedural Characteristics

Characteristic	Overall	Group N	Group D	*P* value
	*N* = 54	*N* = 43	*N* = 11	
Age, years	62.2 (13.7)	61.9 (14.2)	63.6 (12.0)	.71
Male, *n*	40 (74%)	32 (74%)	8 (73%)	1.00
Body surface area, m^2^	1.7 (0.2)	1.7 (0.2)	1.7 (0.2)	.62
Hypertension, *n*	33 (61%)	27 (63%)	6 (55%)	.73
Diabetes mellitus	6 (11%)	6 (14%)	0 (0%)	.33
Dyslipidaemia, *n*	17 (31%)	15 (35%)	2 (18%)	.47
Left ventricular end-diastolic diameter, mm	52.9 (5.9)	53.5 (5.9)	50.3 (5.5)	.11
Left ventricular end-systolic diameter, mm	34.2 (4.3)	34.6 (4.4)	32.9 (4.1)	.26
Left ventricular ejection fraction, %	64.1 (4.5)	64.3 (4.2)	63.3 (5.6)	.51
Surgical approach, *n*				.50
Median sternotomy	31 (57%)	26 (60%)	5 (45%)	
Right thoracotomy	23 (43%)	17 (40%)	6 (55%)	
Procedure, *n*				
Resection	40 (74%)	32 (74%)	8 (73%)	1.00
Neochordae	22 (41%)	16 (37%)	6 (55%)	.32
Edge to edge	8 (15%)	8 (19%)	0 (0%)	.18
Folding	2 (3.7%)	2 (4.7%)	0 (0%)	1.00

Continuous variables were expressed as mean (standard deviation). Categorical variables were expressed as number (percentage).

### Anatomical parameters of the mitral valve by TEE

In the overall cohort, the anteroposterior and transverse diameters were 33.2 (4.3) mm and 36.3 (4.2) mm, respectively (**Table 2**). The anteroposterior diameter and perimeter of the mitral annulus were not different between group D and group N, while the transverse diameter was significantly larger in group D than in group N. The position of the anterolateral commissure was often near 65 degrees counterclockwise from the anteroposterior axis, and the position of the P1-P2 cleft was often near 125 degrees. These positions were similar between the groups. MAD was most frequently observed at P1, and all patients in group D had disjunction at P1.

**Table 2.** Anatomical Parameters of the Mitral Valve by Transoesophageal Echocardiography

	Overall	Group N	Group D	*P* value
	*N* = 54	*N* = 43	*N* = 11	
Anteroposterior diameter, mm	33.2 (4.3)	33.2 (4.2)	33.1 (4.5)	.94
Transverse diameter, mm	36.3 (4.2)	35.6 (3.9)	39.2 (4.5)	.011
Anteroposterior diameter (3D), mm	34.3 (3.7)	34.1 (3.8)	34.8 (3.6)	.58
Transverse diameter (3D), mm	40.4 (4.4)	39.7 (4.4)	43.0 (3.4)	.025
Perimeter, mm	125.1 (11.8)	123.9 (12.2)	130.0 (9.5)	.13
Anterolateral commissure, degrees	65.0 [60.0, 70.0]	65.0 [60.0, 70.0]	65.0 [60.0, 70.0]	.63
Cleft of P1-P2, degrees	125.0 [120.0, 130.0]	125.0 [120.0, 130.0]	125.0 [120.0, 132.5]	.77
Localization of MAD, *n*				
P1	11 (100%)	0 (NA%)	11 (100%)	NA
P2	1 (9.1%)	0 (NA%)	1 (9.1%)	NA
P3	2 (18%)	0 (NA%)	2 (18%)	NA
Minimum ML distance	4.5 (2.1)	4.9 (2.1)	3.2 (1.1)	.012

Continuous variables were expressed as mean (standard deviation) or median [25th and 75th percentiles]. Categorical variables were expressed as a number (percentage).

Abbreviations: MAD: mitral annular disjunction and ML distance: distance between the mitral annulus and the left circumflex coronary artery.

### The distance between the mitral annulus and the LCX

**Figure 3.**
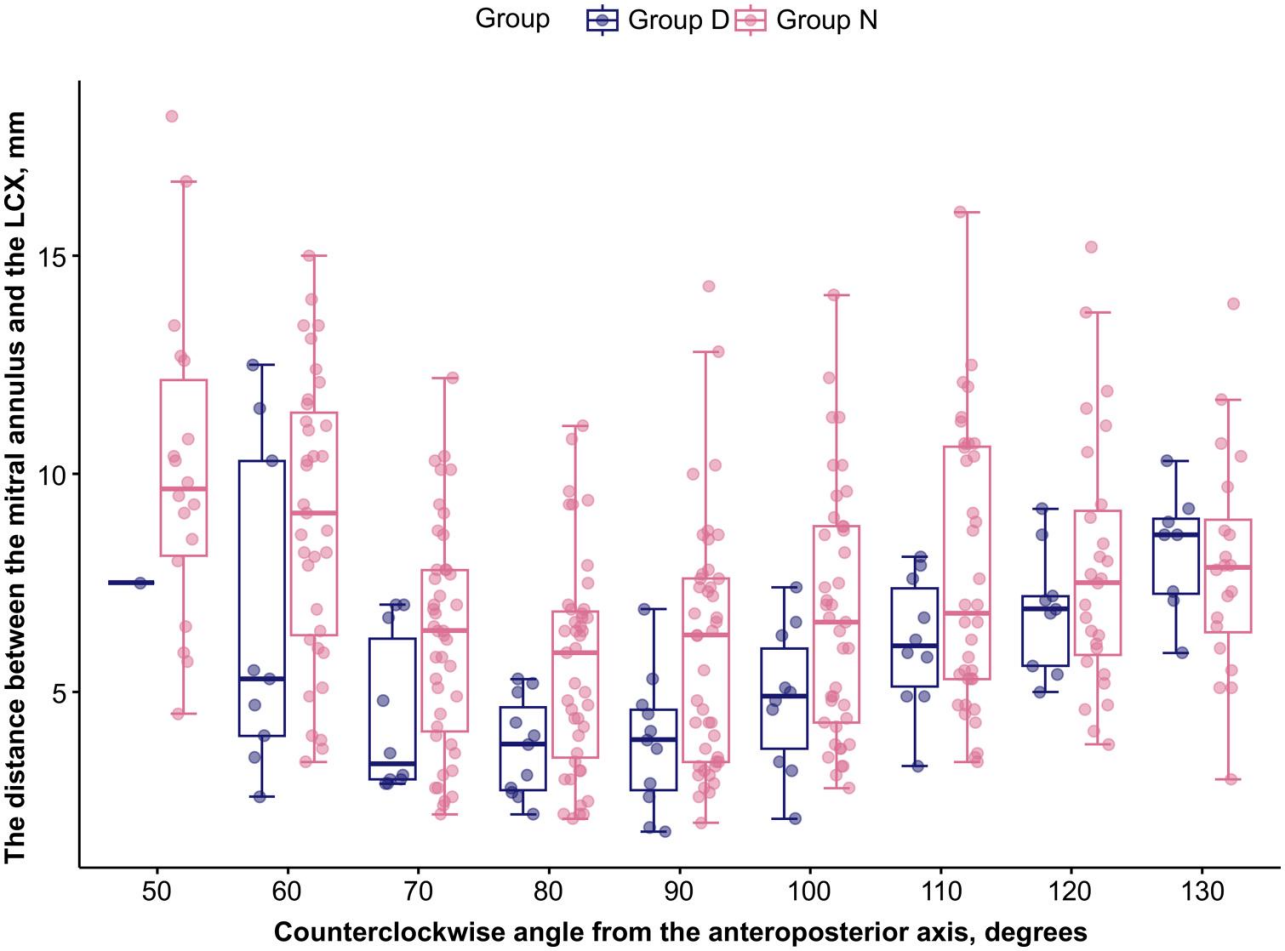
The Distance between the Mitral Annulus and the LCX in Group D and Group N. LCX, left circumflex coronary artery

The LCX was closest to the mitral annulus at 70-90 degrees counterclockwise from the anteroposterior axis (5.9 [2.5] mm at 70 degrees, 5.2 [2.3] mm at 80 degrees, and 5.5 [2.7] mm at 90 degrees). In approximately 15% of the cases, the ML distance was less than 3 mm at 70-90 degrees (**Table 3** and **Figure 3**). In the overall cohort, no cases of LCX injury were observed. The minimum value of ML distance in this series was 1.8 mm in one of the cases in group D.

**Table 3.** The Distance between the Mitral Annulus and the LCX

Angles, degrees	Overall	Group N	Group D	Coefficient	95% CI	*P* value
	*N* = 54	*N* = 43	*N* = 11			
50	10.0 (3.6)	10.1 (3.6)	7.5 (NA)	−2.61	−9.92, 4.71	.49
60	8.5 (3.4)	9.0 (3.2)	6.7 (3.7)	−2.37	−4.81, 0.08	.064
70	5.9 (2.5)	6.2 (2.6)	4.4 (1.8)	−1.84	−3.52, −0.16	.036
80	5.2 (2.3)	5.6 (2.4)	3.7 (1.1)	−1.87	−3.35, −0.39	.017
90	5.5 (2.7)	5.9 (2.8)	3.8 (1.5)	−2.10	−3.84, −0.36	.022
100	6.4 (2.7)	6.7 (2.8)	4.9 (1.6)	−1.89	−3.74, −0.05	.0497
110	7.4 (3.0)	7.7 (3.2)	6.1 (1.5)	−1.58	−3.62, 0.46	.14
120	7.6 (2.6)	7.8 (2.9)	6.9 (1.4)	−0.97	−2.96, 1.03	.35
130	8.0 (2.2)	7.9 (2.5)	8.2 (1.4)	0.35	−1.53, 2.22	.72

Continuous variables were expressed as mean (standard deviation).

Abbreviations: CI: confidence interval and LCX: left circumflex coronary artery.

Overall, the ML distance was shorter in group D than in group N and was significantly shorter at 70-100 degrees (4.4 [1.8] mm versus 6.2 [2.6] mm, *P* = 0.036 at 70 degrees; 3.7 [1.1] mm versus 5.6 [2.4] mm, *P* = 0.017 at 80 degrees; 3.8 [1.5] mm versus 5.9 [2.8] mm, *P* = 0.022 at 90 degrees; and 4.9 [1.6] mm versus 6.7 [2.8] mm, *P* = 0.0497) (**Table 3** and **Figures 2 and 3**). The minimum ML distance was significantly shorter in group D than in group N (3.2 [1.1] mm versus 4.9 [2.1] mm, *P* = 0.012) (**Table 2**).

Regarding the association between the ML distance and the coronary bed anatomy, the distance was significantly shorter in the balanced and left dominant groups compared to the right dominant group at 70-90 degrees (**Table 4**).

**Table 4.** The Distance between the Mitral Annulus and the LCX according to the Coronary Bed Anatomy

Angles, degrees	Balanced	Left dominant	Right dominant	*P* value
	*N* = 3	*N* = 5	*N* = 46	
50	9.2 (1.7)	7.7 (2.0)	10.6 (3.9)	.47
60	6.0 (2.3)	6.7 (2.8)	9.0 (3.5)	.15
70	3.5 (2.0)	4.2 (1.5)	6.2 (2.5)	.047
80	2.7 (0.8)	3.2 (1.2)	5.6 (2.3)	.012
90	2.7 (0.6)	3.5 (1.3)	5.9 (2.7)	.029
100	4.0 (0.6)	4.5 (0.8)	6.8 (2.8)	.067
110	4.9 (0.4)	5.5 (1.3)	7.8 (3.0)	.085
120	6.1 (1.2)	5.9 (1.6)	8.0 (2.7)	.15
130	7.6 (0.4)	6.8 (2.4)	8.3 (2.3)	.38

Continuous variables were expressed as mean (standard deviation).

Abbreviation: LCX: left circumflex coronary artery.

## DISCUSSION

The main findings of this study were as follows: (1) Intraoperative TEE combined with software-based MPR images is less invasive and useful for studying the relationship between the mitral annulus and the LCX. (2) The LCX was closest to the mitral annulus at 70-90 degrees counterclockwise from the anteroposterior axis (at P1 near the anterolateral commissure). (3) The distance between the mitral annulus and the LCX was significantly shorter at 70-100 degrees in patients with MAD than in patients without MAD. We could avoid LCX injury during mitral valve surgery using MPR of intraoperative TEE for measurement of the ML distance and placing annuloplasty sutures with attention to the risk of LCX injury, though no comparative study was performed. Furthermore, few reports exist regarding the association of MAD with the proximity of the LCX to the mitral annulus. Our results from the exploratory analysis suggested the association between MAD and short ML distance, though further studies with a larger sample size would be needed to confirm this possibility.

LCX injury is a rare complication during mitral valve surgery, and the incidence has been reported to be as high as 1.8%.^7^ One of the reasons for this complication is that the LCX may run a course close to the mitral annulus. Various reports have appeared in the literature about the modalities for visualizing the relationship between the mitral annulus and the LCX. Ender et al reported the usefulness of intraoperative TEE in visualizing the LCX.^8^ It is reliable and routinely performed in cardiac surgery, though it is operator-dependent. Ghersin et al studied the three-dimensional relationship between the mitral annulus and the coronary arteries using cardiac CT scans.^9^ This modality was more reproducible and could visualize the LCX at a wider angle. However, contrast-enhanced CT cannot be performed for all patients for reasons such as chronic kidney disease and an allergy for contrast agents. We used the post-processed MPR images obtained from intraoperative TEE to study the relationship between the mitral annulus and the LCX. This method is more reproducible than TEE alone and can accurately identify the mitral annulus (leaflet attachment) and the LCX, as it can be played as a video.

Our data showed that the closest proximity area of the LCX to the mitral annulus was at a 70-90 degree counterclockwise angle from the anteroposterior axis, which corresponds to P1 near the anterolateral commissure. Our results are compatible with previous studies.^2^^,^^9^^,^^10^ Furthermore, we showed that the ML distance was shorter, especially in patients with MAD than in patients without MAD, and that this tendency is pronounced around 70-100 degrees in our model. Our results suggested the possibility of the anatomical association between the ML distance and MAD, though it was an exploratory analysis. Amongst patients with mitral valve prolapse, the prevalence of MAD has been reported to vary between 20% and 58%.^11^ We included only patients who underwent mitral valve repair for degenerative mitral regurgitation, and almost all of them had mitral valve prolapse. Therefore, the 20% prevalence of MAD in our cohort was consistent with the previous reports. It has been reported that MAD was located most frequently at P1 and P2, and less commonly at P3.^12^^,^^13^ Indeed, in our study, all patients with MAD had disjunction at P1. Although the reason for the association between MAD and short ML distance remains unclear, we believe there may be an embryological predisposition. Kishimoto et al^3^ also identified MAD and left coronary dominance as factors related to the proximity between the mitral annulus and LCX. In this study, left coronary dominance was also associated with proximity between the mitral annulus and LCX.

Though several possible mechanisms of LCX injury are possible: direct laceration of the vessel, complete encircling by a suture, and distortion due to tissue retraction,^14^ recognizing the ML distance seems to be helpful in order to avoid LCX injury. MPR of intraoperative TEE images would be helpful to identify patients with a short ML distance. No cases of LCX injury were observed in our study, and we could not identify the distance associated with a high risk of LCX injury. However, according to the previous reports, LCX injuries were observed in patients with an ML distance of 3-3.5 mm.^15^ In our study, approximately 15% of the patients had an ML distance less than 3 mm at 70-90 degrees. To prevent the LCX injury, it is important to place sutures towards the left ventricle and avoid deep sutures near P1 during annuloplasty, especially in patients with a short ML distance. Furthermore, when MAD or left coronary dominance is recognized preoperatively, surgeons can anticipate a high-risk anatomy for LCX injury and place sutures cautiously.

Our study has several limitations. First, the subgroup analysis according to the presence or absence of MAD was exploratory, and the small number of patients enrolled in this study limits our conclusion regarding the association between the ML distance and MAD. Also, observer bias may be present, as recognition of MAD could occur at the same time as measurement of the distance, though measurements were performed before investigating the association between MAD and short ML distance. Confirmation in a larger series with more robust imaging modalities, such as CT scans, would be needed. Second, measurement of the distance by TEE is limited in the systolic phase due to the limited range of the echo beam. According to the report by Ghersin et al, the distance between the mitral annulus and the LCX is similar between different phases of the cardiac cycle.^9^ Third, because measurements were performed by a single experienced observer, interobserver agreement could not be assessed. Also, while there appears to be no major deviation compared to previous reports,^2^^,^^9^ we would evaluate external validity in future studies. Fourth, measurements of the distance by TEE have a technical limitation. It is difficult to measure the distance in patients with an extremely large mitral annulus because the LCX falls outside the visible range of TEE.

## CONCLUSIONS

MPR of intraoperative TEE images is a less invasive and useful tool to detect patients with a short ML distance. The area of the closest distance from the mitral annulus to the LCX is at P1 near the anterolateral commissure, especially in patients with MAD.

## Data Availability

The data underlying this article will be shared on reasonable request to the corresponding author.
